# Phytoestrogens and risk of prostate cancer: a meta-analysis of observational studies

**DOI:** 10.1186/s12957-015-0648-9

**Published:** 2015-07-31

**Authors:** Jinjing He, Shuai Wang, Mi Zhou, Weiwen Yu, Yuelong Zhang, Xiang He

**Affiliations:** Department of Urology, Affiliated Zhejiang Provincial People’s Hospital, School of Medicine, Zhejiang University, Shangtang Road 158, Hangzhou, 310014 Zhejiang Province People’s Republic of China; Department of Ophthalmology, Affiliated Zhejiang Provincial People’s Hospital, School of Medicine, Zhejiang University, Hangzhou, People’s Republic of China

**Keywords:** Phytoestrogens, Prostate cancer, Meta-analysis, Observational studies, Serum concentration

## Abstract

**Background:**

Epidemiologic studies have reported various results relating phytoestrogens to prostate cancer (PCa). The aim of this study was to provide a comprehensive meta-analysis on the extent of the possible association between phytoestrogens (including consumption and serum concentration) and the risk of PCa.

**Methods:**

Eligible studies were retrieved via both computer searches and review of references. The summary relative risk ratio (RR) or odds ratio (OR) and 95 % confidence interval (CI) were calculated with random effects models.

**Results:**

A total of 11 studies (2 cohort and 9 case–control studies) on phytoestrogen intake and 8 studies on serum concentration were included in the meta-analysis. The pooled odds ratio (OR) showed a significant influence of the highest phytoestrogens consumption (OR 0.80, 95 % CI 0.70–0.91) and serum concentration (OR 0.83, 95 % CI 0.70–0.99) on the risk of PCa. In stratified analysis, high genistein and daidzein intake and increased serum concentration of enterolactone were associated with a significant reduced risk of PCa. However, no significant associations were observed for isoflavone intake, lignans intake, or serum concentrations of genistein, daidzein, or equol.

**Conclusions:**

The overall current literature suggests that phytoestrogen intake is associated with a decreased risk of PCa, especially genistein and daidzein intake. Increased serum concentration of enterolactone was also associated with a significant reduced risk of PCa. Further efforts should be made to clarify the underlying biological mechanisms.

## Background

Prostate cancer (PCa) was the second most frequently diagnosed cancer and the sixth leading cause of death from cancer among men worldwide in 2008 according to the estimate of the International Agency for Research on Cancer [1]. The worldwide PCa burden is expected to grow to 1.7 million new cases and 499,000 new deaths by 2030 simply due to the growth and aging of the global population [2]. Given the 25-fold variation in disease incidence between population at the highest and lowest risk [1], lifestyle, diet, environmental, and genetic factors have been suggested to play a role in the etiology of the disease [3, 4]. The association between dietary factors and PCa has been investigated and one explanation for the low incidence of the cancer in Asia might be high consumption of soybeans and its products [5, 6], which are rich in one class of phytoestrogens known as isoflavones.

Phytoestrogens, which have structural and functional similarities to 17b-oestradiol, are believed to have a prophylactic effect on PCa [7]. There are 3 main classes of phytoestrogens: isoflavones, lignans, and coumestans. In Western populations with a low intake of isoflavones, phytoestrogen intake is predominantly derived from intake of plant lignans. It is reported that isoflavones, lignans, and their metabolites have anticarcinogenic properties [8, 9]. Isoflavones principally include genistein, daidzein, and glycitein. Equol is a metabolite of daidzein produced by the intestinal microflora [10] that has higher oestrogenic activity than its parent isoflavone. The most abundant lignan in human subjects is enterolactone, which is produced by certain types of intestinal microflora from plant lignan glycosides. Variation in individual metabolism of phytoestrogens due to differences in gut microflora [11] may influence the serum concentration of phytoestrogens and their biologic effects. It is reported that the capacity to produce equol has been found be to lower among American than Japanese and Korean men [12]. So, it is important to quantify the association between serum concentration of phytoestrogens and risk of PCa.

According to 2 previous meta-analyses [13, 14], consumption of soy products rich in isoflavones are inversely associated with PCa risk. However, both of them focused on the soy consumption neglecting the intake of plant lignans (which is the primary phytoestrogen intake in Western populations). Meanwhile, neither one evaluated the association between serum concentration of phytoestrogens and risk of PCa.

Therefore, we performed a meta-analysis to address this gap. We updated and assessed quantitatively the association between intake of isoflavones and lignans and risk of PCa from the cohort and case–control studies. We also investigated the association between serum concentration of phytoestrogens and their metabolites and the risk of PCa.

## Methods

### Search strategy

We identified relevant publications in the MEDLINE database using PubMed, Web of Science, and the Cochrane Library up to June 2014. Search terms included “phytoestrogens,” “isoflavones,” “lignans,” “flavonoids,” “genistein” or “daidzein,” “glycitein,” “equol,” “enterolactone,” and “enterodiol,” combined with “prostate cancer” or “prostatic carcinoma”. Two of the authors (SW and JH) reviewed the titles and abstracts independently to exclude any clearly irrelevant studies. The full texts of the remaining articles were read to determine whether they contained information on the topic of interest. Any disagreements were resolved by discussion. Furthermore, references in the retrieved publications, as well as those in previous reviews [15, 16], were checked for any other pertinent studies.

### Study selection

To be included, studies had to fulfill all of the following inclusion criteria: (i) case–control or cohort study published as an original article reported in English between 1980 and February 2014, (ii) estimated the relationship between phytoestrogens (intake or serum concentration) and the risk of PCa, (iii) provided a risk estimate relative risk (RR) [or odds ratio (OR)] and its 95 % confidence intervals (CI) or sufficient information allowing us to compute them, and (iv) adjustment made for age and potential risk factors. In studies with overlapping patients or controls, only the latest or the most informative were included. Any study with inconsistent or erroneous data was excluded. Meeting abstracts with insufficient data or unpublished reports were not considered. We included all methods for measuring exposure to phytoestrogens such as questionnaires, interviews, and serum level or urinary excretion. We did not include studies that used tumor-related biomarkers (such as PSA) as outcome. We also exclude data concerning phytoestrogens and the risk of recurrent PCa.

### Data extraction

For each study, the following characteristics were extracted: last name of first author, publication year, country in which the study was conducted, study design, population type and sample size, adjustment for potential confounders, definition of phytoestrogens exposure status, and estimates of associations. The levels of phytoestrogens exposure varied considerably among the studies, so we extracted the most adjusted risk estimate of the highest reported category of phytoestrogens exposure relative to the lowest from these studies for comparison.

### Statistical analyses

The ORs were used as the common measure of association across studies by considering the RRs as ORs. The data from individual studies were pooled by use of the random effects model with the DerSimonian-Laird method [17], which considers within-study and between-study variation. We performed subgroup analyses based on different kinds of phytoestrogens. Certain items, such as biochanin A, coumestrol, secoisolariciresinol, or matairesinol, were seldom assessed in individual reports; these analyses were not performed. Meta-regression analysis was used to assess the heterogeneity in publication year, study conducted area, study design, and sample size. The Q-statistic and I^2^ score were used to assess the between-study heterogeneity of results [18, 19]. Publication bias assessment was done using the Egger regression asymmetry test [20] and the Begg-adjusted rank correlation test [21]. If publication bias was observed, the “trim and fill” method [22] was used to calculate an estimate of the effect size after considering publication bias (adjusted effect size). Possible outliers were visually identified and tested for their effect on the significance of the effect size. The statistical software used was Stata/SE 11.0 (Stata Corporation, College Station, TX), and the significance level was set to *P* < 0.05.

## Results

### Phytoestrogen intake

**Fig. 1:**
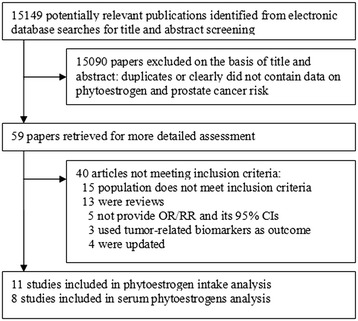
Study selection process

The detailed steps of our literature search are shown in Fig. 1. We identified 13 studies that had investigated the association between on phytoestrogen intake and the PCa risk. We excluded two studies [23, 24] from the analysis because they were updated by Hedelin et al. [25] and Word et al. [26]. The remaining 11 studies selected for analysis are presented in Table 1. Two were cohort studies [27, 28], and the other nine were case–control studies. All of them used quantitative food frequency questionnaire (FFQ) to measure phytoestrogen intake. Two of these studies were quintile comparisons [27, 29], six were quartile comparisons [25, 28, 30–33], and three reported comparison between populations with low and high intakes [26, 34, 35]. Of the studies, four were conducted in North America, four in Europe, and three in Asia. Because different kinds of phytoestrogens were evaluated in these studies, some of which assessed more than one kind of phytoestrogen, we chose the risk estimate for the phytoestrogen kind that was representative of their phytoestrogen intake in overall analysis. These phytoestrogen kinds were prioritized in descending order of total phytoestrogens or isoflavones, genistein, daidzein, and lignans. Subsequently, we did the stratified analysis of individual types of phytoestrogens.

**Table 1:** Epidemiologic studies on phytoestrogen intake in association with prostate cancer risk

Reference	Study site/race	Design	Cases/controls or cohort size	Dietary assessment	Phytoestrogens	Contrast	Adjusted OR (95 % CI)	Adjustment
Park et al. [27] 2008	USA/multiethnic	Cohort	4404/82,483	QFFQ (118 items)	Genistein	<0.7 vs. ≥3.1 mg/1000 kcal	0.94 (0.84–1.04)	Time since cohort entry, ethnicity, family history of prostate cancer, education level, BMI, smoking status, and energy intake
					Daidzein	<0.7 vs. ≥3.2 mg/1000 kcal	0.92 (0.82–1.02)	
					Total isoflavones	<1.6 vs. ≥7.2 mg/1000 kcal	0.93 (0.83–1.04)	
Kurahashi et al. [28] 2007	Japan/Japanese	Cohort	307/43,509	FFQ (147 items)	Genistein	<13.2 vs. ≥32.8 mg/day	0.71 (0.48–1.03)	Age, area, smoking status, drinking frequency, marital status, BMI, intake of total fatty acids, dairy, vegetables, and fruits
					Daidzein	<8.5 vs. ≥20.4 mg/day	0.77 (0.52–1.13)	
Nagata et al. [31] 2007	Japan/Japanese	HCC	200/200	Semi-quantitative FFQ	Isoflavones	<30.5 vs. ≥89.9 mg/day	0.48 (0.25–0.93)	Smoking, energy, and PUFA intake
					Genistein	<1.1 mg/day vs. ≥ 2.5 mg/day	0.68 (0.39–1.20)	
					Daidzein	<0.8 mg/day vs. ≥1.9 mg/day	0.64 (0.36–1.17)	
Heald et al. [32] 2007	Scotland/Scottish	PCC	433/483	SCG-FFQ	Isoflavones	≤581.1 μg/day vs. ≥1982.8 μg/day	1.18 (0.79–1.75)	Age, total energy intake, family history of PCa and BrCa, Carstairs Deprivation Index, smoking and energy intake: BMR ratio
Bosetti et al. [29] 2006	Italy/Italian,	HCC	1294/1451	FFQ	Isoflavones	≤14.7 vs. ≥32.2 μg/day	0.98 (0.76–1.26)	Terms for age, study center, education, body mass index, family history of prostate cancer, and total calorie intake
Hedelin et al. [25] 2006	Sweden/Swedish	PCC	1499/1130	FFQ (261 items)	Phytoestrogens	≤1.18 vs. >4.71 μg/day	0.74 (0.57–0.95)	Age, intake of antibiotics, zinc, animal fat, total energy intake, alcohol, vegetable fat, red meat during the last year
					Lignans	≤113 vs. >213 μg/day	0.85 (0.65–1.12)	
					Isoflavonoids	≤1.0 vs. >2.6 μg/day	0.99 (0.77–1.28)	
					Genistein	≤0.27 vs. >1.08 μg/d	0.97 (0.75–1.26)	
					Daidzein	≤0.49 vs. >1.11 μg/d	1.22 (0.92–1.62)	
Lee et al. [30] 2003	China/Chinese	HCC	133/265	FFQ	Genistein	<17.9 vs. >62.0 mg/day	0.53 (0.29–0.97)	Age and total calories
					Daidzein	<10.0 vs. >36.3 mg/day	0.56 (0.31–1.04)	
Strom et al. [34] 1999	USA/American white	HCC	83/107	FFQ (modified block)	Genistein	Low vs. high	0.71 (0.39–1.30)	Age, family history of prostate cancer, alcohol intake, and total caloric intake
					Daidzein		0.57 (0.31–1.05)	
McCann et al. [33] 2005	USA/American	PCC	433/538	FFQ (172 items)	Lignans	<335.4 vs. >603.9 μg/day	0.66 (0.47–0.94)	Age, education, body mass index, cigarette smoking status, and total energy
Word et al. [26] 2010	UK/British Caucasians	Nested C-C	203/800	FFQ and 7-day food diaries	Daidzein	Low vs. high	0.88 (0.72–1.09)	Age, height, weight, physical activity, social class, family history of prostate cancer, and daily intake of energy, fat, zinc, selenium, dairy products, and lycopene
					Genistein		0.89 (0.72–1.09)	
					Total isoflavones		0.87 (0.70–1.09)	
					Total lignans		0.96 (0.71–1.31)	
Lewis et al. [35] 2009	USA/American	HCC	478/382	Block FFQ (100 items)	Genistein	≤196.0 vs. >196.0 mcg	0.54 (0.33–0.89)	Age, education, BMI, smoking history, family history of prostate cancer in first-degree relatives, and total caloric intake
					Daidzein	≤77.0 vs. >77.0 mcg	0.54 (0.33–0.89)	

*QFFQ* quantitative food frequency questionnaire, *PUFA* polyunsaturated fatty acid, *SCG-FFQ* Scottish Collaborative Group-FFQ, *EPIC* European Prospective Investigation into Cancer and Nutrition, *HCC* hospital-based case–control, *PCC* population-based case–control, *BMI* body mass index, *PCa* prostate cancer

**Fig. 2:**
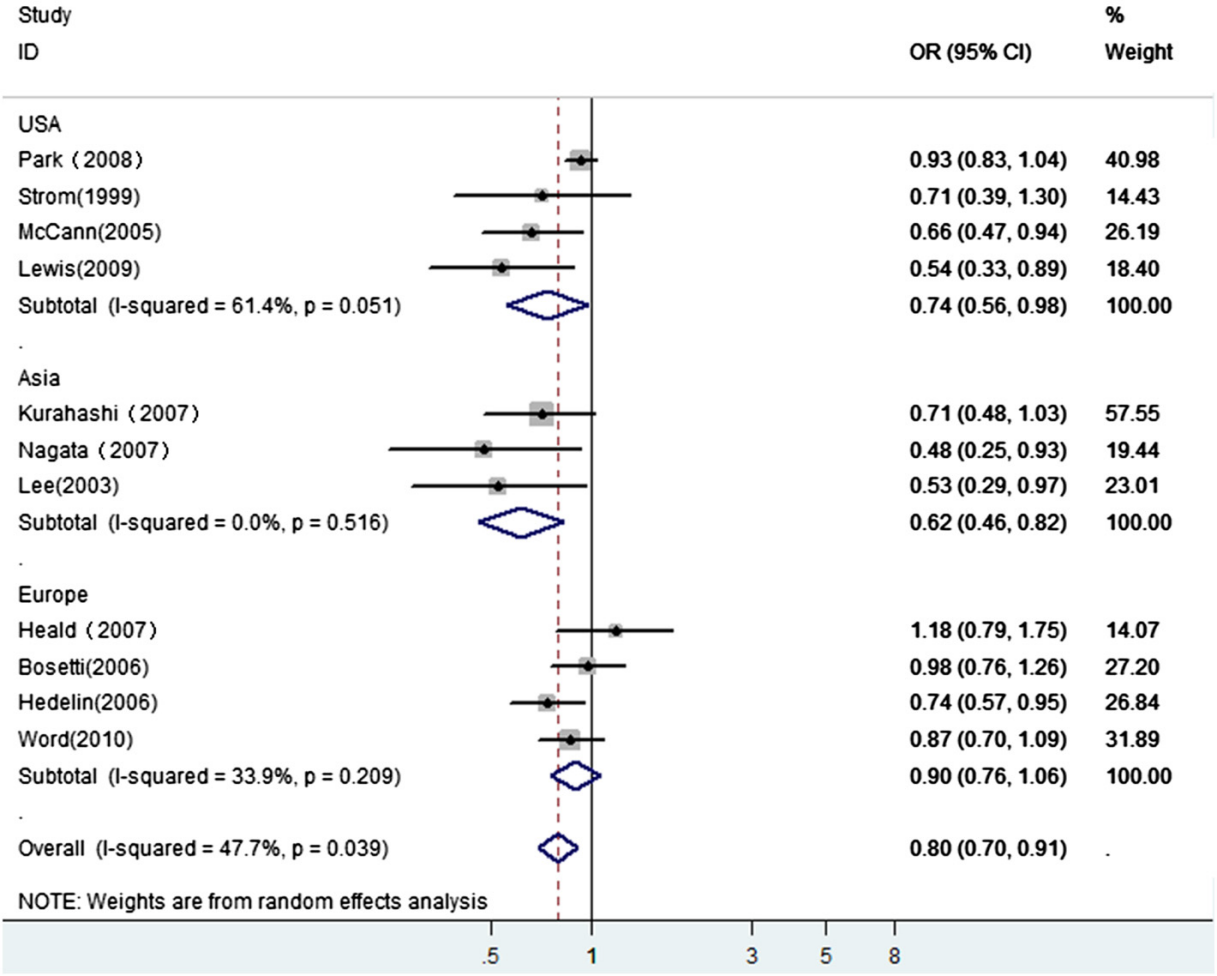
A forest plot showing pooled data for the association between phytoestrogen intake and prostate cancer risk in a variety of geographical region

We found that phytoestrogen intake (OR 0.80, 95 % CI 0.70–0.91) was statistically significantly associated with reduced risk of PCa with significant heterogeneity (I^2^ = 47.7, *P* = 0.039). We used meta-regression analysis to explore the influence of publication year, study design, sample size, and study conducted area. However, none was identified as a possible source of heterogeneity among all the included studies (data not shown). Further scrutiny found that the heterogeneity was reduced when the analysis was stratified by geographical region (see Fig. 2). The association was stronger among Asian (OR 0.62, 95 % CI 0.46–0.82) than American (OR 0.74, 95 % CI 0.56–0.98) and European (OR 0.90, 95 % CI 0.76–1.06). The funnel plot showed some asymmetry. Begg’s test (*P* = 0.043) and Egger’s test (*P* = 0.021) for publication bias were significant. The trim and fill analysis yielded the same conclusions without evidence of any potentially missed unpublished studies.

**Fig. 3:**
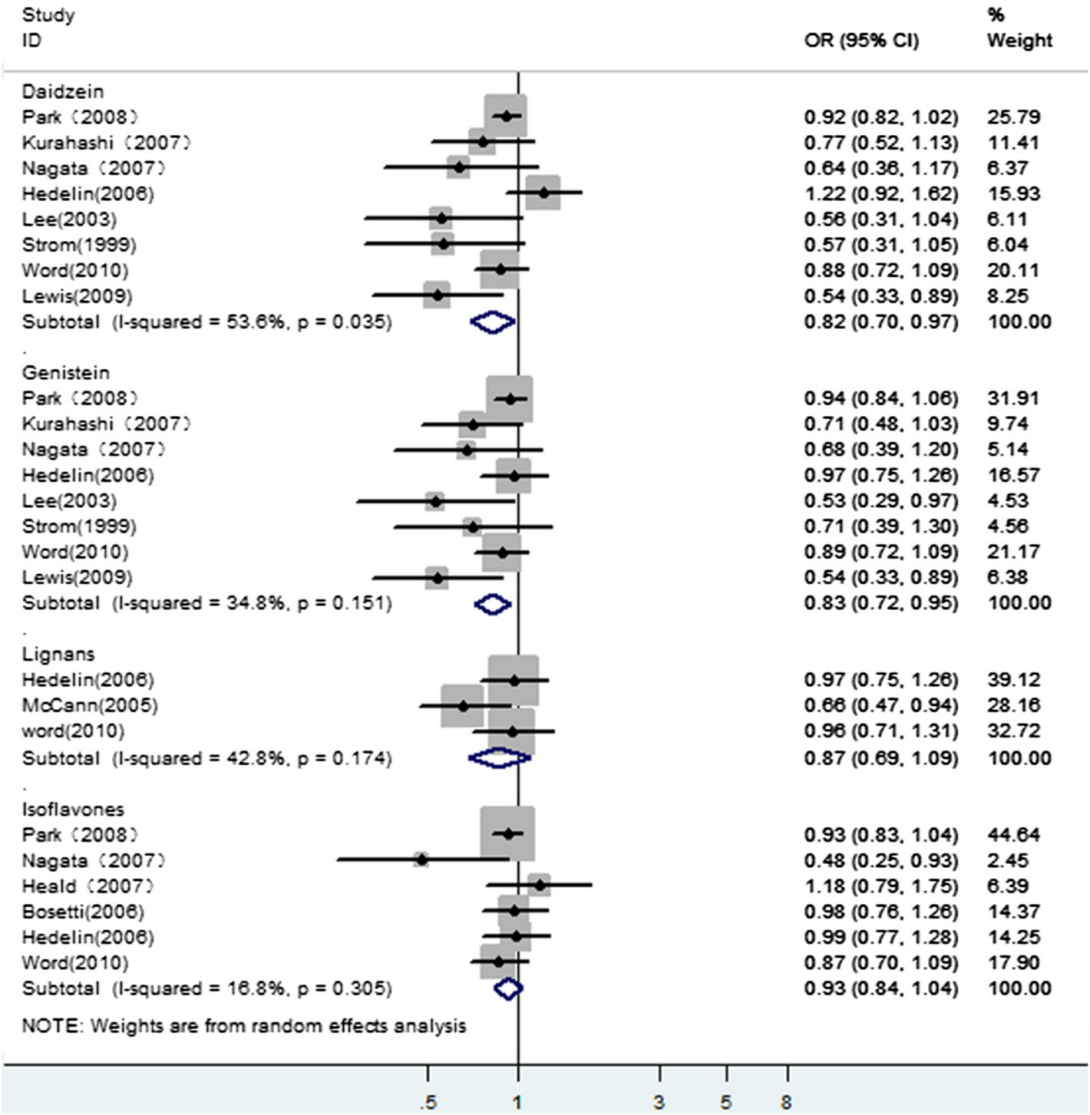
A forest plot showing the pooled risk estimates of prostate cancer for different types of phytoestrogen intake

In stratified analysis of individual types of phytoestrogens (see Fig. 3), eight studies investigated the genistein and daidzein, three studies tested lignans, and six studies tested the isoflavones. The risk of PCa decreased significantly in association with high consumption of genistein (OR 0.83, 95 % CI 0.72–0.95) and daidzein (OR 0.82, 95 % CI 0.70–0.97), but high consumption of lignans (OR 0.87, 95 % CI 0.69–1.09) and isoflavones (OR 0.93, 95 % CI 0.84–1.04) were not significantly associated with the risk of PCa. Heterogeneity was detected (*P* = 0.035) among the eight studies evaluating daidzein intake and the risk of PCa. In contrast, there was no evidence of heterogeneity among studies of genistein, lignans, and isoflavones.

The Begg’s tests and Egger’s test provided no evidence of publication bias for daidzein and isoflavones (data not shown). However, for genistein analysis, the Begg’s tests indicated no substantial publication bias (*P* = 0.083), while the Egger’s test provided evidence for publication bias (*P* = 0.005). We used the trim and fill method to estimate the missing studies. The result yielded the same conclusions without evidence of any potentially missed unpublished studies.

### Serum phytoestrogens

**Fig. 4:**
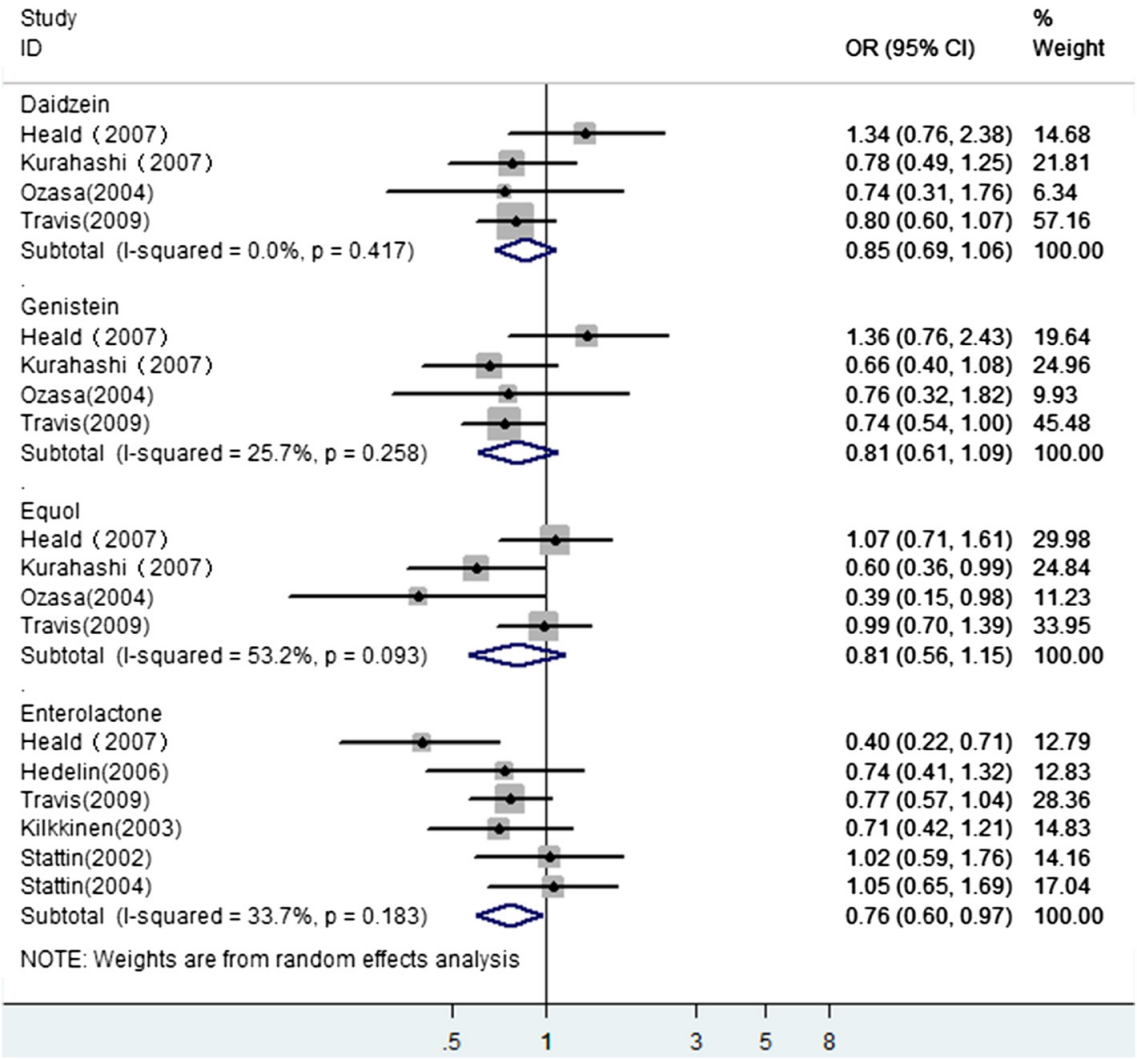
A forest plot depicting the pooled risk estimates on the association between serum phytoestrogen concentration and prostate cancer risk

We identified 10 studies that had investigated the association between serum phytoestrogen concentration and the risk for PCa. We excluded two studies [26, 36] because they presented results identical to those of a later publication. One study [37] contained data from three countries that were recently updated for two of these countries [38, 39]. So, we included all these three studies and extracted the latest data. The remaining 8 studies selected for analysis are presented in Table 2. All of them are case–control studies. Five of these studies are quartile comparisons [25, 32, 37–39], two are tertile comparison [40, 41], and one is a quintile comparison [42]. Four studies investigated genistein, daidzein, and equol [32, 40–42]. Six studies investigated the plasma enterolactone [25, 32, 37–39, 42]. Only one study provided the relationship between serum isoflavones and PCa risk [32]. In overall combination of serum phytoestrogens, these serum phytoestrogen kinds were prioritized in descending order of total isoflavones, genistein, daidzein, equol, and enterolactone. The summary OR was 0.83 (95 % CI 0.70–0.99), and the *P* value for heterogeneity was 0.525. The Begg funnel plots were symmetric, and the Egger’s tests provided no evidence of publication bias (*P* = 0.497). When stratified analysis was conducted of individual types of serum phytoestrogens, only serum enterolactone was inversely associated with the risk of PCa with no heterogeneity (*P* = 0.183) (see Fig. 4). High serum concentration of genistein, daidzein, and equol were not associated with the risk of PCa (see Fig. 4). There was no heterogeneity among these subgroup studies. No publication bias was detected either by Begg’s test or by Egger’s test in all subgroups (data not shown).

**Table 2:** Epidemiologic studies on serum phytoestrogens concentrations in association with prostate cancer risk

Reference	Study site/race	Design	Cases/controls	Serum phytoestrogens	Contrast	Adjusted OR (95 % CI)	Adjustment
Heald et al. [25] 2007	Scotland/Scottish	PCC	249/205	Equol	0 vs. ≥0.10 nmol/l	1.07 (0.71–1.61)	Age, total energy intake, family history of PCa and BrCa, Carstairs Deprivation Index, smoking and energy intake: BMR ratio.
				Daidzein	≤8.26 vs. >29.11 nmol/l	1.34 (0.76–2.38)	
				Genistein	≤14.23 vs. >64.53 nmol/l	1.36 (0.76–2.43)	
				Enterolactone	≤8.41 vs. >28.90 nmol/l	0.40 (0.22–0.71)	
Hedelin et al. [32] 2006	Sweden/Swedish	PCC	1499/1130	Enterolactone	≤15.2 vs. >37.8 nmol/l	0.74 (0.41–1.32)	Age, intake of antibiotics, zinc, animal fat, total energy intake, alcohol, vegetable fat, red meat during the last year
Kurahashi et al. [40] 2007	Japan/Japanese	NCC	307/43,509	Genistein	<57 vs. ≥151.7 ng/ml	0.66 (0.40–1.08)	Smoking status, alcohol intake, marital status, and intake of green tea, protein, fiber, and green or yellow vegetables.
				Daidzein	<22 vs. ≥61.5 ng/ml	0.78 (0.49–1.25)	
				Equol	<1.0 vs. ≥15.0 ng/ml	0.60 (0.36–0.99)	
Ozasa et al. [41] 2004	Japan/Japanese	NCC	52/151	Genistein	<239 vs. >682 nM	0.76 (0.32–1.82)	Age
				Daidzein	<89 vs. >239 nM	0.74 (0.31–1.76)	
				Equol	<1.9 vs. >56.1 nM	0.39 (0.15–0.98)	
Travis et al. [42] 2009	EPIC	NCC	950/1042	Genistein	<0.30 vs. ≥7.00 ng/ml	0.74 (0.54–1.00)	Smoking, education, BMI, physical activity, alcohol intake, and marital status
				Daidzein	<0.30 vs. ≥4.10 ng/ml	0.80 (0.60–1.07)	
				Equol	<0.05 vs. ≥0.80 ng/ml	0.99 (0.70–1.39)	
				Enterolactone	<0.05 vs. ≥0.80 ng/ml	0.77 (0.57–1.04)	
Kilkkinen et al. [38] 2003	Finland	NCC	214/214	Enterolactone	<5.9 vs. ≥24.4 nmol/l	0.71 (0.42–1.21)	Age match
Stattin et al. [37] 2002	Norway, Finland, Sweden	NCC	794/2550	Enterolactone	<8.9 vs. ≥27.89 nmol/l	Finland 1.21 (0.91–1.60)	Age match
					<3.49 vs. ≥11.58 nmol/l	Norway 1.02 (0.59–1.76)	
					<7.15 vs. ≥25.14 nmol/l	Sweden 0.87 (0.45–1.67)	
Stattin et al. [39] 2004	Sweden	NCC	265/525	Enterolactone	<9.38 vs. ≥28.31 nmol/l	1.05 (0.65–1.69)	Age, BMI, smoking, and fasting

*EPIC* European Prospective Investigation into Cancer and Nutrition (include 23 centers in 10 European countries), *BMI* body mass index, *NCC* nested case–control, *PCC* population-based case–control, *PCa* prostate cancer

## Discussion

This meta-analysis demonstrated that consumption of phytoestrogens was associated with a reduction in PCa risk of 20 % in men when the highest reported intake was compared with the lowest reported intake. The results of our separate analysis based on the type of phytoestrogens showed inverse associations for the consumption of genistein and daidzein and with increased serum concentrations of enterolactone. However, no significant associations were observed for isoflavone intake, lignan intake, or serum level of genistein, daidzein, and equol.

It has been suggested that phytoestrogens may prevent cancer by a variety of mechanisms, including sex and/or growth hormone regulation, antioxidant properties, apoptosis of PCa cells, and/or inhibition of angiogenesis, invasion, and metastasis [43, 44]. These bioavailable metabolites can be estimated from dietary intake data by using in vitro data from incubation of foods with human feces [45, 46], but this assessment does not take into account interindividual variations in microbial synthesis. Moreover, the quantification of the phytoestrogen content of food can vary threefold to fourfold, depending on variety, environmental factors, growth, harvesting time, and processing [47]. So, the associations between phytoestrogen intake and risk of PCa were not so convincing. Measurement of metabolites in blood and urine is considered to be more objective and precise [48]. As we all know, human gut microflora have been shown to exert metabolic activities on phytoestrogens [49]. As the gut microflora may differ by its concentration and composition from one person to the other, antimicrobials (i.e., antibiotics) may lead to intra- and interindividual variations in amounts of intestinal phytoestrogen metabolites that are converted from consumed phytoestrogens by the gut bacteria. In comparison to the metabolism of isoflavones, biotransformation of lignans has been found less variable [50]. This may explain why we found a significant association with increased serum concentrations of enterolactone but did not find significant associations with serum concentrations of genistein, daidzein, and equol. Of course, that may be also related to other bioactive isoflavone metabolites that were not researched yet.

Due to the potential benefits, there have been six randomized controlled trials [51–56] that used phytoestrogens in man already diagnosed with PCa. Most of them had small sample sizes and were of short duration. These studies assessed the effect of soy/isoflavones on tumor marker (prostate-specific antigen, PSA) or hormonal markers levels in men with PCa. So far, no study was reported on 5-year survival or metastasis. A search of ClinicalTrials.gov reveals numerous currently ongoing trials that are investigating the role of phytoestrogens in the treatment of PCa (i.e., NCT01126879, NCT01325311, NCT01682941, NCT01036321, NCT00345813). The results of those studies will provide more evidence for the role of phytoestrogens.

It is worth noting that in our analysis, stratification by region yielded a significant inverse association with PCa risk for studies in Asian populations, a marginally significant inverse association with risk for studies in US populations but no significant association with risk for those in Europe populations. This difference may, in part, be due to differences in environment and dietary patterns in these regions. Characterization of the individual variability as defined by the gut microflora composition and gene polymorphisms [23] may also help to explain the discrepancies observed so far.

As a meta-analysis of previously published observational studies, our study has several limitations that need to be taken into account. First, only English language articles were included. We did not attempt to uncover unpublished observations and did not include studies with insufficient information to estimate an adjusted OR, which could bring publication bias, even though the trim and fill analysis yielded the same conclusions without evidence of any potentially missed unpublished studies. Second, for now, the two available cohort studies could not provide sufficient data for meta-analysis, so we have to choose case–control studies as the data resource for analysis. In addition, because some items were combined only from three studies, the total numbers of cases remained low. Third, the intake levels of phytoestrogens in the lowest and highest categories and the range of consumption level varied across studies. Moreover, food frequency questionnaires which assess dietary habits may lead to measurement errors. It is not only due to recall bias but also to the estimation by using different food composition databases, which may not be complete for the whole range of foods consumed. On the other hand, variation in individual metabolism of phytoestrogens due to differences in gut microflora and measurement in a single blood or urine sample may only reflect recent dietary intake. These differences may have contributed to the heterogeneity among studies. To be honest, phytoestrogen spectrum and content varies between the plant species, sort, and origin. Even the same molecule arising from the different sources can exert various effects. It may not be excluded that synthetic phytoestrogens with desirable structure and activity could be an easier and safer alternative of the traditional plant product of variable origin, phytoestrogen content, and activity. Several studies are going on developing novel and more selective synthetic phytoestrogens.

## Conclusions

Our findings support the hypotheses that serum enterolactone and consumption of genistein and daidzein protect against PCa risk. Interestingly enough, an association between PCa risk and isoflavone intake or serum concentrations of its metabolites was not found. The complexity of phytoestrogen composition and its metabolism make the evaluation of the effect of phytoestrogen on PCa very difficult. In the light of these findings, further prospective epidemiological studies using improved food databases and experimental studies are needed to identify the specific compounds that provide protection, to determine precisely how the complex metabolism of phytoestrogens may interact with other mechanisms to prevent cancer. Synthetic phytoestrogens with desirable structure and activity could be an easier and safer alternative of the traditional plant product.

## Abbreviations

PCa: prostate cancer
RR: risk ratio
OR: odds ratio
CI: confidence interval
PSA: prostate-specific antigen
FFQ: quantitative food frequency questionnaire
